# Effect of social media use on the orthorexia nervosa tendency among pregnant women

**DOI:** 10.12669/pjms.41.6.12008

**Published:** 2025-06

**Authors:** Pelin Calpbinici

**Affiliations:** 1 Pelin Calpbinici, PhD, RN Assistant Professor in Dept. of Obstetrics & Gynecology Nursing, Nevşehir Hacı Bektaş Veli University, Semra and Vefa, Kucuk Faculty of Health Sciences, Nevsehir, Turkiye

**Keywords:** Eating Behavior, Health Behavior, Orthorexic, Pregnancy, Social Media

## Abstract

**Objective:**

This study was conducted to examine the effects of social media use in pregnant women on orthorexia nervosa (ON) tendency.

**Methods:**

The descriptive study was carried out between June 10 and September 30, 2022 with 200 pregnant women admitted to the Gynecology and Obstetrics Clinic. The researchers’ Information Form, the Healthy Eating Obsession Scale (ORTO-11), and the Social Media Use Integration Scale (SMUIS) were used to collect data.

**Results:**

The mean ORTO-11 scale score of pregnant women was 29.18 ± 4.52 and the mean SMUIS scale score was 29.37 ± 10.02. A very weak, negative, statistically significant relationship was found between the mean score of the pregnant women from the SMUIS scale and the mean score from the ORTO-11 scale (p<0.05). According to multiple linear hierarchical regression analysis, age (β=-0.183; p<0.05), place of residence (β=0.181; p<0.05), intended pregnancy (β=0.140; p<0.05), paying attention to the food intake (β=0.139; p<0.05), BMI (β=0.151; p<0.05) and, SMUIS (β=0.829; p<0.05) were significant predictors of the ON tendency in pregnant women.

**Conclusion:**

In our study, it was found that the use of social media by pregnant women increased their tendency to develop orthorexia nervosa. These findings are important in terms of interventions for the detection, prevention, and treatment of orthorexia nervosa during pregnancy.

## INTRODUCTION

The term “Orthorexia Nervosa” (ON) was first introduced in 1997 in an article published in the Yoga Journal by Steven Bratman. ON is an obsessive-compulsive disorder characterized by excessive care and selection of foods considered “healthy”. ON is briefly defined as “an unhealthy obsession with healthy eating”,^1^ yet it remains absent from the DSM-5, so no consensus diagnostic criteria exist.^2^ Nevertheless, clinical studies have identified hallmark features of ON,^3^ notably its focus on food quality rather than quantity.^4^ Individuals with ON rigidly avoid items containing artificial colors, sweeteners, preservatives, pesticide residues, genetically modified ingredients, unhealthy fats, or excessive salt and sugar.^4^,^5^ Beyond meal preparation, they devote substantial time to researching, cataloging, weighing, and measuring foods, as well as planning future meals. This phobic avoidance of “unhealthy” foods intensifies over time: diets become stricter, and deviations provoke guilt, fear of illness, and further self-punishing restrictions.^3^,^5^ As a result, ON can lead to malnutrition, emotional instability, social isolation, and depressive symptoms.^3^,^6^ Prevalence estimates underscore its potential impact: approximately 6.5% of university students exhibit orthorexic tendencies,^7^ a rate that soars to 32.1–81.8% in high-risk groups such as artists, athletes, and healthcare professionals,^8^,^9^ and reaches 26.6% among pregnant women.^5^

Pregnancy is an important period for the onset, recurrence, or recovery of eating disorders.^10^ Overall, a woman’s sense of responsibility during pregnancy is multifaceted. By embracing this responsibility, a pregnant woman aims to ensure the best possible outcomes for herself and her baby. This responsibility can cause significant anxiety in the mother, leading to an obsession with nutrition.^5^ Accordingly, ON in pregnancy can be defined as the pregnant woman’s obsession with healthy eating due to the thought of not harming her baby. Irregular nutrition during pregnancy is associated with numerous negative consequences such as spontaneous abortion, premature birth, low birth weight infants, increased C-section delivery, and other obstetric and postpartum difficulties.^11^ Therefore, it is necessary to ensure adequate and balanced energy and nutrient intake during pregnancy to protect the health of the mother and fetus.^12^

In recent years, social media platforms have emerged as powerful shapers of dietary norms, with “clean eating” and wellness communities proliferating across Instagram, TikTok, and Facebook.^13^ These communities frequently promote increased consumption of fruits and vegetables and reduced intake of processed foods—but can also amplify disordered eating by normalizing extreme dietary restrictions, ideological purity tests, and fear-based messaging.^14^ For pregnant women—already navigating heightened concerns about fetal health—exposure to idealized “healthy pregnancy” feeds may intensify orthorexic tendencies.^15^ Yet, despite this plausible link, the specific impact of social media use on ON tendency during pregnancy remains unexamined. To address this gap, our study employed two validated instruments. ON tendency was assessed with the Turkish version of the Healthy Eating Obsession Scale (ORTO-11).^16^-^18^ Social media engagement was measured using the Social Media Use Integration Scale (SMUIS), a 10 items instrument developed by Jenkins-Guarnieri et al.^19^, which captures both emotional connection and behavioral integration of social media into daily life.

Building on these robust measurement foundations and acknowledging the lack of prior research in this area, our study was designed to explore pregnant women’s social media usage patterns, quantify their ON tendencies, and determine whether—and to what extent—social media integration predicts orthorexic behaviors during pregnancy. Such insights have important clinical implications, as they may enable obstetric and nutrition professionals to screen for problematic social media habits in prenatal care and to develop targeted interventions that promote balanced, evidence-based eating during this critical period. This study aimed to examine the effect of social media use on ON tendency in pregnant women, specifically the following:

- What are the social media usage characteristics of pregnant women?
- What is the ON tendency level in pregnant women?
- Does the use of social media by pregnant women affect ON tendency levels?

## METHODS

This descriptive study was conducted with pregnant women who presented to the Obstetrics and Gynecology Clinic of Nevsehir State Hospital in Turkey. A simple random sampling technique was employed: from all eligible pregnant women meeting the inclusion criteria, 200 were randomly selected and invited to participate; those who consented were enrolled. In the post-study power analysis (G-Power 3.1.9.7) to calculate the sample power, it was calculated that the sample had an effect size of 0.38 with a 95% confidence interval and 99% power.

### Inclusion criteria:

- Voluntary participation in the study.
- No communication problem such as vision and speech.
- No psychiatric problem.
- No fetal anomaly.
- Gestation for 12 to 40 weeks; and being a social media user.

### Exclusion criteria:

- Chronic systemic diseases known to affect appetite or dietary behavior (e.g. diabetes mellitus, thyroid disorders, celiac disease)
- High-risk obstetric complications (e.g. preeclampsia, gestational diabetes, placenta previa)

### Ethical considerations:

Written approval (Date: 28.03.2022, Issue No: 2022.03.24) was obtained from the Non-Interventional Ethics Committee of Nevşehir Hacı Bektaş Veli University. Written permission (Date: 30.05.2022, Issue No: E-26171210-929) was obtained from the Nevşehir State Hospital management in order to conduct the study.

### Measurements

Information Form, Healthy Eating Obsession Scale (ORTO-11)^17^,^18^ and Social Media Usage Scale (SMUIS)^19^,^20^ were used to collect data.

### Information form:

The Information Form was prepared by the researchers in line with the relevant literature.^4^,^5^ It contained 21 questions about the socio-demographic and obstetric characteristics of women and six questions about their social media use (including social networks used and their frequency, purpose of seeking information, subjects researched, belief in the reliability of information in social media, and whether the information causes anxiety, attention to food intake, weight satisfaction, and attention to diet).

### Healthy Eating Obsession Scale (ORTO-11):

The ORTO-15 was developed by Donini et al.^17^ to measure the healthy eating obsession in individuals. The validity and reliability of this scale in the Turkish population were confirmed by Arusoğlu et al.,^18^ and it was adapted into Turkish as ORTO-11 after the elimination of items with factor loads of 0.50 and below. The answers which had distinguishing criteria for orthorexia were given “1” point, while the answers which showed normal eating behaviours tendency were given “four” points. Items indicative of orthorexia are scored as “one,” whereas those reflecting normative eating behaviors are scored as “four.” Total scores range from 11 to 44, with values below 27 % considered indicative of orthorexia.^17^ All items are scored in the same direction except item 8, which is reverse-coded. In the original validation study, the ORTO-15 demonstrated a Cronbach’s α of 0.70.^17^ Following item reduction, the Turkish ORTO-11 exhibited a Cronbach’s α of 0.62 in Arusoğlu et al.’s adaptation, and in our study, Cronbach’s α was 0.64.

### Social Media Use Integration Scale (SMUIS):

The original scale was developed by Jenkins-Guarnieri, Wright & Johnson.^19^ The scale is a the 6-point Likert type, with a maximum score of 60 and minimum of 10. The scale has no cut-off point. High scores indicate increased levels of social media usage. The scale consists of two sub-dimensions: Social Integration and Emotional Connection; and Integration with Social Routines. The Cronbach’s alpha value of the original scale was 0.89 for the Social Integration and Emotional Connection sub-dimension, 0.83 for the Integration with Social Routines sub-dimension, and 0.91 for the whole scale.^19^ The adaptation of the scale to Turkish was carried out by Akin et al.^20^ The Cronbach’s alpha internal consistency reliability coefficients of the Turkish version of the scale were reported as 0.87 for the Social Integration and Emotional Connection sub-scale, 0.71 for the Social Routine Integration subscale, and 0.87 for the whole scale.

### Data collection:

Face-to-face interviews were used to collect data between June 10 and September 30, 2022. The interview took 15 minutes. Although 245 pregnant women who met the sampling criteria came to the polyclinic at the time of the study, 45 pregnant women did not want to participate in the study, and the study was completed with 200 pregnant women.

### Data analysis:

Statistical analyses were performed using the Statistical Package for the Social Sciences (SPSS) software, version 25.0 (IBM Corporation, Armonk, New York, United States). For categorical measurements, numbers and percentages were used, whereas means and standard deviation were used for numerical measurements. The conformity of the scales to the normal distribution in terms of variable levels was determined by the “Kolmogorov-Smirnov” and “Shapiro-Wilk” tests according to the number of samples. The relationship between the scales was evaluated by using the Pearson Correlation Coefficient. The Mann Whitney U Test was used to compare the differences between the use of social media sites by pregnant women and their ORTO-11 score averages, since the data did not show normal distribution. Multiple linear regression analysis was used to investigate the association of age, body mass index (BMI), and social media use levels with ON tendency in pregnant women. The statistical significance level was set at p<0.05.

## RESULTS

The mean age was 26.18 ± 5.55 years and 40% of the pregnant women were aged 18–24 years. Additionally, 38% of the pregnant women were high school graduates; 81.5% were housewives; 75.0% had nuclear families; 72.5% reported that their perceived income was equal to their expenses; 62% were multiparous, and 77.0% were between 25 and 40 weeks of gestation. Overall, 78% women stated that it was a wanted pregnancy and 22.5% had received prenatal education. The mean BMI was 26.89 ± 4.39, and the BMI of 40.0% women was 25.0–29.9 kg/m^2^. Moreover, 52.0% women were satisfied with their weight; 58.5% paid attention to their diet at a normal level; 69.5% ate 3–4 meals a day; 84.5% paid attention to the foods they bought; and 59.0% planned their meals in advance (Table-I).

**Table-I:** Distribution of socio-demographic, obstetric and nutritional characteristics of the pregnant women (N=200).

Descriptive characteristics	n	%	ORTO-11 (Mean±SD)	Statistical Analysis	p-Value
***Age (years)***					
18-24	88	44.0	29.88±4.35	3.367	0.037**
25-30	73	36.5	29.16±4.75		
31-40	39	19.5	27.64±4.21		
***Educational status***					
Elementary	68	34.0	30.10±4.43	2.431	0.091**
High school	76	38.0	28.95±4.42		
University and higher	56	28.0	28.38±4.67		
***Employment***					
Not employed (Housewife)	163	81.5	29.33±4.69	1.154	0.253*
Employed	37	18.5	28.51±3.68		
***Family type***					
Extended	150	25.0	29.54±4.53	0.648	0.518*
Nuclear	50	75.0	29.06±4.54		
***Perceived income level***					
Income is less than expenses	31	15.5	29.35±5.85	9.076	0.011**
Income is equal	145	72.5	29.54±4.27		
Income is more than expenses	24	12.0	26.75±3.44		
***Place of Residence***					
City	99	49.5	28.29±4.57	4.843	0.009**
County	41	20.5	29.31±4.30		
Village/Town	60	30.0	30.55±4.31		
***Parity***					
Primiparous	76	38.0	28.98±4.78	-0.471	0.638*
Multiparous	124	62.0	29.29±4.39		
***Week of pregnancy***					
14-24	46	23.0	28.37±4.64	-1.386	0.167*
25-40	154	77.0	29.42±4.48		
***Intended pregnancy***					
Yes	156	78.0	28.83±4.51	-2.094	0.038**
No	44	22.0	30.43±4.40		
***Participation inparental education classes***					
Yes	45	22.5	28.77±4.30	-0.676	0.500*
No	155	77.5	29.29±4.59		
***BMI***					
Underweight (<18.5 kg/m^2^)	2	1.0	30.00±2.82	8.055	0.045 ***
Normal (18.5–24.9 kg/m^2^)	72	36.0	28.29±4.20		
Overweight (25.0–29.9 kg/m^2^)	80	40.0	29.09±4.72		
Obese (≥30.0 kg/m^2^)	46	23.0	30.69±4.45		
***Weight satisfaction***					
Satisfied	104	52.0	29.11±4.29	2.455	0.293***
Not Satisfied	78	39.0	29.62±4.92		
Not sure	18	9.0	27.72±3.97		
***Pay attention to diet***					
Very Careful	16	8.0	27.63±4.72	4.682	0.182***
Normal level	117	58.5	29.07±4.63		
A little attention	54	27.0	29.20±3.93		
Don’t pay any attention	13	6.5	31.92±5.02		
***Number of meals per day***					
1-2	32	16.0	27.53±5.16	4.872	0.088***
3-4	139	69.5	29.49±4.43		
5 and more	29	14.5	29.48±3.97		
***Paying attention to the foods intake***					
Yes	169	84.5	28.80±4.39	-2.782	0.006*
No	31	15.5	31.23±4.77		
***Planning the food***					
Yes	118	59.0	28.82±4.56	-1.344	0.181*
No	82	41.0	29.69±4.45		
***Consuming fast food***					
Yes	100	50.0	29.22±4.33	0.125	0.901*
No	100	50.0	29.14±4.74		
***Consuming junk food***					
Yes	151	75.5	29.29±4.45	0.610	0.543*
No	49	24.5	28.83±4.78		

* Independent sample t-test,

** ANOVA,

*** Kruskal Wallis test.

It was found that 50.5% of the pregnant women used social media between 1-3 hours a day, and the most frequently used social media sites were WhatsApp with 95.5% and Instagram with 82.0%. Regarding the purpose of use, 78.5% of women used social media to follow the baby’s development; 54.5% to research birth-related issues; and 47.5% to get information about nutrition during pregnancy. Furthermore, 42% of women stated that social media partially affected their personal decisions; 63.5% found the information on social media partially safe; and 57.0% stated that the information obtained from social media partly worried them.

The mean score of the participants in the SMUIS scale was 29.37 ± 10.03; the mean scores of the Social Integration and Emotional Attachment, and Social Routines Integration sub-dimensions were 14.97±6.49 and 14.40±4.49, respectively. The mean score of the ORTO-11 was 29.18±4.52. A very weak, negative, statistically significant correlation was found between the mean scores of the SMUIS and ORTO-11 scales (r=-0.145; p<0.05). As the SMUIS score average increased very weakly, the ORTO-11 score average decreased very weakly.

A very weak, negative, statistically significant correlation was found between the mean score of the Social Integration and Emotional Attachment sub-dimension of the SMUIS scale and the ORTO-11 scale mean score (r=-0.178; p<0.05). Accordingly, as the average score of the Social Integration and Emotional Attachment sub-dimension increased very weakly, the ORTO-11 score average decreased very weakly (Table-II).

**Table-II:** The Relationship Between Pregnants’ Social Media Use Levels and Orthorexia Nervosa Tendency (n=200).

SMUIS and Sub-dimension	Score (Mean ± SD)	ORTO-11 (Mean ± SD)	
		29.18 ± 4.52	
		r	p
SMUIS total score	29.37 ± 10.03	-0.145	0.040*
Social Integration and Emotional Attachment	14.97 ± 6.49	-0.178	0.012*
Integration with Social Routines	14.40 ± 4.49	-0.068	0.341

* p < 0.05, r=Pearson correlation coefficient.

The results of the multiple linear hierarchical regression analysis are displayed in Table-III. Variables that were statistically significant in bivariate analysis (age, perceived income level, place of residence, intended pregnancy, paying attention to food intake, BMI, and SMUIS) were included in the multiple linear hierarchical regression analysis. First, control variables (age, perceived income level, place of residence, intended pregnancy, paying attention to food intake, and BMI) were included in the model. Then, the SMUIS variable was also included in the second model. The overall contribution of the variables included in the first model was significant (F=5.464, p<0.001) and these variables explained 14.5% of the variance.

**Table-III:** Hierarchical regression analysis predicting orthorexia nervosa tendency in pregnant women (n=200).

Independent variables	Model 1			Model 2		
	B	SE	β	B	SE	β
Constant	21.846	2.774	-	24.849	2.963	-
Age	-1.088	0.423	-0.183*	-1.284	0.424	-0.216*
Income level	-0.257	0.600	-0.030	-0.293	0.591	-0.034
Place of Residence	0.938	0.357	0.181*	0.858	0.353	0.165*
Intended pregnancy	1.525	0.756	0.140*	1.612	0.758	0.148*
Paying attention to the foods intake	1.741	0.846	0.139*	1.838	0.835	0.147
BMI	0.156	0.070	0.151*	0.143	0.069	0.139*
SMUIS TS				-0.074	0.035	-0.176*
F	5.464			5.809		
p	p<0.001			p<0.001		
R^2^	0.145			0.175		
ΔR^2^	0.119			0.145		

* p<0.05; Dependent Variable: ORTO-11; SE: Standard Error; BMI: Body Mass Index.

In the first model, age (β=-0.183; p<0.05), place of residence (β=0.181; p<0.05), intended pregnancy (β=0.140; p<0.05), paying attention to the food intake (β=0.139; p<0.05), and BMI (β=0.151; p<0.05) were significant predictors of the ON tendency in pregnant women, while place of residence (β=-0.034 p>0.05) did not contribute significantly. After addition of the SMUIS variable in the second model, the variables explain 17.5% of the variance (F=5.809; p<0.05). Accordingly, increasing level of social media usage (β=0.829; p<0.05) was associated with an increase in the level of ON tendency. There was no difference in ON tendency levels between those who used Facebook, Twitter, YouTube, and WhatsApp social media sites and those who did not (p>0.05). In contrast, the increase in the ON tendency levels among pregnant women who used Instagram and TikTok was statistically significant (p<0.05).

## DISCUSSION

Social media has become one of the easiest, most convenient, simply accessible, and time-saving ways for young reproductive-aged women to obtain health information before and during pregnancy.^21^ In our study, 50.5% of pregnant women repoted using social media for one to three hours per day. Similarly, Lee JY et al. and Lee E et al. found that pregnant women used social media to obtain pregnancy information an average of 21.94±22.94 times a week.^22^ Pregnant women use social media to meet their pregnancy-related needs, get social and emotional support, or search for topics they are curious about.^23^ Building on overall use, we examined specific topics of interest. The majority of pregnant women (78.5%) used social media to follow the development of the baby and about half (47.5%) to obtain information about nutrition during pregnancy. Our study result is similar to that of the study by Zhu et al.^24^ who reported that nutrition is the most mentioned topic after fetal development on social media sites. In a systemic review by Sayakhot and Carolan-Olah, it was found that pregnant women most commonly sought information on fetal development, nutrition during pregnancy, drug use during pregnancy, and prenatal care on the internet.^25^ These parallels suggest that, across settings, pregnant women turn to social media for both factual information and reassurance. However, the type of content encountered matters. Turner and Lefevre found that 54% of individuals used social media to explore and share their food experiences, while 42% used social media to get advice about food.^14^ Likewise, Rounsefell et al.’s meta-analysis linked social media exposure—especially to image-based posts—to dieting behaviors, overeating, and selective healthy food choices.^26^ Consequently, the emotional triggers and normative cues embedded in social feeds may subtly influence dietary decisions.^27^

Pregnancy introduces additional vulnerability. As women confront changing bodies and intensifying concerns about fetal well-being, they may be particularly susceptible to “healthy-eating” trends online—such as orthorexia nervosa (ON).^14^,^28^ In our study, the mean ORTO-11 score was found to be 29.37±10.02. Similarly, Öztürk et al. and Şenol et al.^29^ found 29.29±3.77, Taştekin Ouyaba A et al. and Çiçekoğlu Öztürk P et al.^5^ found 29.40±3.80. These consistent findings confirm the presence of orthorectic behaviors among expectant mothers.

Pregnant women are often forced by their social environment to pay too much attention to healthy nutrition and food content.^14^ In addition, they may follow social media accounts on issues related to nutrition during pregnancy. Indeed, in our study, a relationship was found between ON symptoms as measured by the ORTO-11 and social media usage levels, and high levels of social media use were associated with a greater ON tendency.

Furthermore, using Instagram increased the ON tendency among pregnant women. Similar to our study, Turner and Lefevre reported that high Instagram usage is associated with an increased ON tendency.^14^ This situation can be explained as follows: social media users decide for themselves which accounts they want to follow and are constantly exposed to the type of content produced by these accounts, which encourages selective exposure. Limited exposure may lead users to believe this behavior is more common and/or normal than it actually is, and there may be perceived social pressure to comply with such behavior.^14^ Although not seen in every pregnant woman, changes in eating habits during pregnancy are common during pregnancy, and using social media as a guiding source of nutrition may trigger the tendency toward eating disorders in some pregnant women.

We found that the use of TikTok as well as Instagram increased the ON tendency in pregnant women. Minadeo and Pope showed that weight loss was glorified in many posts on TikTok, food was positioned to achieve health and slimness, and there was a lack of experts providing nutritional information.^30^ In the same study, it was determined that 47% of all videos coded under the hashtag “Nutrition” gave some kind of nutritional advice.^30^ TikTok is a common social media platform used by many young adults, and posts about food, nutrition, and weight are very popular. It is crucial to conduct more studies in this area and raise awareness among adults who are at risk, such as pregnant women.

Turning to platform-specific insights, in our study, nearly half of the women (42.0%) stated that social media partially influenced their personal decisions, and the majority of women (63.5%) found the information obtained from social media partially reliable. Our study results support previous findings that women find the information they obtain on the internet and social media safe and use it.^31^ However, the use of social media by pregnant women can have a negative impact on their diet and lifestyle choices. Especially during pregnancy, malnutrition and irregular eating behaviors such as micronutrient, vitamin, and calorie restriction may lead to worrisome health consequences for the mother and fetus.^27^,^28^ Therefore, it is important that health personnel do not ignore the negative aspects of social media and follow pregnant women closely.

### Strengths of the Study:

This study has several notable strengths: by collecting data through face-to-face interviews during routine prenatal visits, we achieved high response completeness and could immediately clarify any participant questions; the use of the validated Turkish ORTO-11 allowed direct comparison with both local and international cohorts; and our platform-specific analysis—separately quantifying Instagram and TikTok engagement and linking each to orthorexia nervosa tendencies—provided novel, granular insights into how different social media channels contribute to disordered eating behaviors.

### Limitations:

Nevertheless, the findings must be interpreted in light of certain limitations. The findings obtained can only be generalized to the region where the study was conducted. The study is limited to the data obtained from pregnant women who agreed to participate in the study on the dates when the data collection tools were applied. The data are based on the statements of the participants and are not clinically validated. The scope of ORTO-11 in identifying ON may be limited due to its psychometric properties. In particular, the ORTO-11 scale is not specific to pregnancy. There is no scale in the literature that assesses orthorexia in pregnancy. Since studies investigating orthorexia nervosa in pregnancy are limited, comprehensive studies on this subject should be planned and conducted.

## CONCLUSION

The results of the study revealed the effect of social media use among pregnant women on their tendency to develop orthorexia nervosa. This is the first study in the literature to examine the effect of social media on the ON tendency in pregnant women. For this reason, it is thought that it will make an important contribution to the literature. It is very important for nurses and midwives working in the prenatal field to take into account the tendency of orthorexia nervosa while monitoring the gestational weight of pregnant women during prenatal follow-up and to detect ON in the early period and to refer them to a specialist. It is recommended that nurses and midwives help pregnant women access the right resources on nutrition and direct them to reliable social media resources.

## Data Availability

The datasets that support the findings of this study are available from the corresponding author upon reasonable request.
